# Modulating the glioma microenvironment with laser interstitial thermal therapy: mechanisms and therapeutic implications

**DOI:** 10.1007/s11060-025-05305-5

**Published:** 2025-11-27

**Authors:** Luis O. Vargas, Vratko Himic, Franciska Otaner, Matthew Abikenari, Jay Chandar, Vaidya Govindarajan, Daniel Kreatsoulas, Arman Jahangiri, Ricardo J. Komotar, Michael E. Ivan, Ashish H. Shah

**Affiliations:** 1 Department of Neurosurgery, University of Buffalo Jacobs School of Medicine and Biomedical Sciences, Buffalo, NY USA; 2 Department of Neurological Surgery, University of Miami Miller School of Medicine, Miami, FL USA; 3 Sylvester Comprehensive Cancer Center, University of Miami Miller School of Medicine, Miami, FL USA; 4 Faculty of Medicine and Health Sciences, McGill University, Montreal, QC Canada; 5 Department of Neurosurgery, Stanford University School of Medicine, Stanford, CA USA

**Keywords:** Glioblastoma, Laser interstitial thermal therapy, Tumor microenvironment, Blood-brain barrier, Immunotherapy, Neuro-oncology

## Abstract

Glioblastoma (GBM) remains one of the most deadly brain tumors through its invasiveness, rapid growth, its immunosuppressive microenvironment, and limited treatment options. Laser interstitial thermal therapy (LITT) is an MR-guided, minimally invasive ablation technique increasingly used in GBM management. This narrative review examines how LITT modulates the glioma microenvironment and explores its therapeutic implications. We cover both preclinical and clinical studies and synthesize the effects of LITT on immune activation, blood-brain barrier (BBB) permeability, and thermal dynamics in gliomas. LITT generates three spatially distinct thermal zones, promoting damage-associated molecular pattern (DAMP) release, immune cell activation, and transient BBB disruption. These changes may help convert immunologically “cold” gliomas into “hot” tumors and enhance the delivery of chemotherapy, immunotherapy, and viral or gene-based therapies. Technical limitations, such as the heat sink effect near vascular structures, are increasingly addressed through innovations like dual-fiber systems and advanced thermal modeling. LITT is emerging as much more than a cytoreductive tool for unresectable glioma; it may provide a platform for immune modulation and therapeutic enhancement in glioma care. Potential benefits of LITT’s interaction with the microenvironment and the BBB include: (1) recruitment and mobilization of the immune system to better target cancerous cells; (2) improved penetration of existing therapies; (3) which enables a lower effective dose for previously barred-drugs, reducing peripheral adverse effects; (4) improved potential for peripheral liquid biopsy. Optimizing treatment timing, patient selection, and combination protocols will be essential to fully harness LITT’s biological effects and improve clinical outcomes.

## Clinical trial number

Not applicable.

## Introduction

Glioblastoma (GBM) is one of the most aggressive and therapeutically challenging CNS malignancies. Despite modern surgery, radiation, and chemotherapy, prognosis remains poor, with median survival rarely exceeding 15 months [1]. A major barrier is tumor location in eloquent or deep-seated regions, where resection risks severe neurological deficits. Furthermore, the glioma microenvironment is immunosuppressive, heterogeneous, and shielded by the blood-brain barrier (BBB), limiting immune infiltration and drug delivery [2].

Laser interstitial thermal therapy (LITT) is a minimally invasive procedure that utilizes a laser fiber probe to deliver heat directly to brain tissue, guided by real-time MRI [3]. As the distance from the fiber probe increases, the temperature gradually decreases, resulting in coagulative necrosis in the tissue surrounding the probe [4]. LITT is approved by the FDA and increasingly utilized in neuro-oncology, with a growing safety record and expanding clinical applications for gliomas and other intracranial pathologies such as radiation necrosis and certain brain metastases. LITT is valuable for treating tumors in surgically inaccessible regions such as the thalamus, insula, and basal ganglia, where resection carries a high risk of neurological morbidity [5]. Across pooled nonrandomized series in eloquent or deep lesions, LITT has been linked to high rates of cytoreduction and low perioperative morbidity; however, variability in patient selection and methodology limits direct comparisons with open surgery. Consistent with this trend, a systematic review/meta-analysis of 17 LITT glioma series (*n* = 401) reported pooled OS of approximately 13.6 months and PFS of around 5 months, supporting feasibility but emphasizing the need for controlled studies trials [6]. Reported benefits (shorter hospital stays, quicker resumption of therapy) should be interpreted cautiously until randomized or carefully matched studies are available. Beyond local tumor ablation, LITT may also modify the tumor microenvironment (TME), used interchangeably with microenvironment, open the BBB, and enhance systemic therapies. Recent evidence synthesized across 39 studies supports LITT as a minimally invasive alternative in deep/inaccessible lesions, with favorable safety and disease-control metrics in tumors, radiation necrosis, and mesial temporal lobe epilepsy [7].

This review explores how LITT modulates the glioma microenvironment via immune activation, BBB permeability, and thermal dynamics. We discuss how these mechanisms can improve therapeutic delivery, potentiate immunotherapy, and overcome barriers posed by gliomas. Understanding these effects is essential to optimizing treatment timing, patient selection, and future treatment protocols. We first examine the TME and heat dissipation in brain tissue, then apply these principles to LITT, specifically ablation, heatsink effects, and transient BBB opening for post-operative drug delivery to the CNS.

While preclinical studies have shown strong immunologic and drug-delivery effects following LITT, clinical validation is still early. Most evidence comes from small prospective studies, pilot cohorts, or case reports, and significant gaps remain between animal models and human glioblastoma. Therefore, this review combines current mechanistic insights and initial clinical signals to propose testable hypotheses rather than definitive treatment claims.

We conducted a targeted narrative review of English-language studies in PubMed, Scopus, and ClinicalTrials.gov, hand-searching reference lists. We included preclinical, translational, and clinical reports on LITT thermal biology and monitoring, TME and immune effects, BBB and BTB modulation and drug delivery, and human LITT outcomes and combination therapies; comparators (e.g., focused ultrasound) were included for context. We excluded non-cranial LITT without generalizable biology and non-English, engineering-only, or irrelevant pediatric reports. Evidence was narratively synthesized as a hypothesis-generating review; no meta-analysis or formal risk-of-bias assessment was performed, and all figures are schematic illustrations.

## The tumor microenvironment

GBM serves as a model for studying TME dynamics. Glioma cells interact with the TME, which promotes tumor growth, spread, and treatment resistance [8]. The TME enables GBM to acquire and sustain oncogenic traits defined in the *Hallmarks of Cancer.* Originally proposed by Hanahan and Weinberg [9], the hallmarks initially described six fundamental biological capabilities acquired during malignant transformation. These include sustaining proliferative signaling, evading growth suppressors, resisting cell death, enabling replicative immortality, inducing angiogenesis, and activating invasion and metastasis. Later updates added traits such as deregulated energetics, immune evasion, genomic instability, tumor-promoting inflammation, plasticity, epigenetic reprogramming, microbiome effects, and senescent cells [10]. These capabilities sustain tumor survival and progression, actively supported by the TME. As such, the immunosuppressive TME plays a dominant role in helping the tumor evade immune destruction and resist therapeutic interventions.

The TME includes cancer-associated fibroblasts (CAFs) [11]. Adaptive and innate immune cells infiltrate the tumor, but often promote its progression [12]. Although cytotoxic CD8⁺ T-cells can trigger anti-tumor immune responses, their activity is inhibited by regulatory T cells (Tregs) and immunosuppressive cytokines such as IL-10 and TGF-β. Tumor-associated macrophages (TAMs) dominate the TME, ranging from pro-inflammatory M1 to immunosuppressive M2 phenotypes. However, it is increasingly recognized that the M1/M2 dichotomy is an oversimplified binary; instead, myeloid cells in GBM display a range of mixed and adaptable activation states that respond to local cytokine signals [13]. This complex myeloid landscape includes not only tumor-associated macrophages (TAMs) and resident microglia but also myeloid-derived suppressor cells (MDSCs) and neutrophils, all of which are now understood to contribute to the immunosuppressive TME [14]. TAMs are often skewed toward an M2-like phenotype that promotes tumor growth and suppresses immune responses. This ‘cold’ phenotype, with sparse T-cell infiltration and high immunosuppression, helps explain why immune checkpoint inhibitors show limited effectiveness in GBM [2]. M2-like TAMs secrete VEGF, stimulating abnormal angiogenesis that fuels tumor growth but impairs drug delivery and T-cell infiltration [15].

The TME also includes non-cellular elements such as the extracellular matrix (ECM), signaling molecules, and extracellular vesicles (EVs). These components provide structural support, influence signaling pathways, and enhance immune suppression. The ECM, composed of collagen and fibronectin, is remodeled to increase stiffness, promote invasion, and raise pressure, hindering drug delivery. Meanwhile, a complex mixture of growth factors, cytokines, and chemokines, such as VEGF, IL-6, IL-10, and CCL2, facilitates crosstalk between tumor and stromal cells, promoting angiogenesis and immunosuppression [16]. Glioblastoma-derived EVs carrying microRNAs, PD-L1,2 and oncogenic proteins transport immunosuppressive cargo to immune cells elsewhere, dampening systemic responses before they reach the tumor. EVs are also useful biomarkers; importantly, interventions like LITT, which disrupt tumor tissue and increase vascular permeability, may enhance the release and detectability of such EVs in circulation, offering a diagnostic window through liquid biopsy [17].

The TME is now seen as an adaptable ecosystem that evolves with the tumor and dictates treatment outcomes. In GBM, this adaptability is particularly evident in the temporal and spatial heterogeneity of the microenvironment. For example, TME composition can differ between the tumor core and edge, or shift with radiotherapy or immunotherapy [18].

Single-cell transcriptomics has revealed distinct immunosuppressive populations, hybrid phenotypes, and dynamic shifts in the TME. GBM myeloid cells often express mixed M1/M2 signatures and switch phenotypes in response to cues [14]. This plasticity highlights the need for temporally precise interventions, such as combining therapies with LITT at immunologically primed windows. Metabolic crosstalk in the TME drives immune suppression. GBM cells and M2-like TAMs compete with T cells for nutrients, while metabolites like lactate and kynurenine suppress T-cell function and expand regulatory cells [27]. Targeting these checkpoints is a major area of investigation, particularly in combination with LITT.

The role of the central nervous system’s unique immune privilege is also being re-examined. Immune cells, including macrophages and T cells, can infiltrate gliomas but often become dysfunctional due to chronic TME-derived suppression. The meningeal lymphatic system aids antigen drainage and T-cell activation, suggesting restoring communication with peripheral immunity could enhance responses [19].

## Thermal zones and immune activation in LITT

**Fig. 1:**
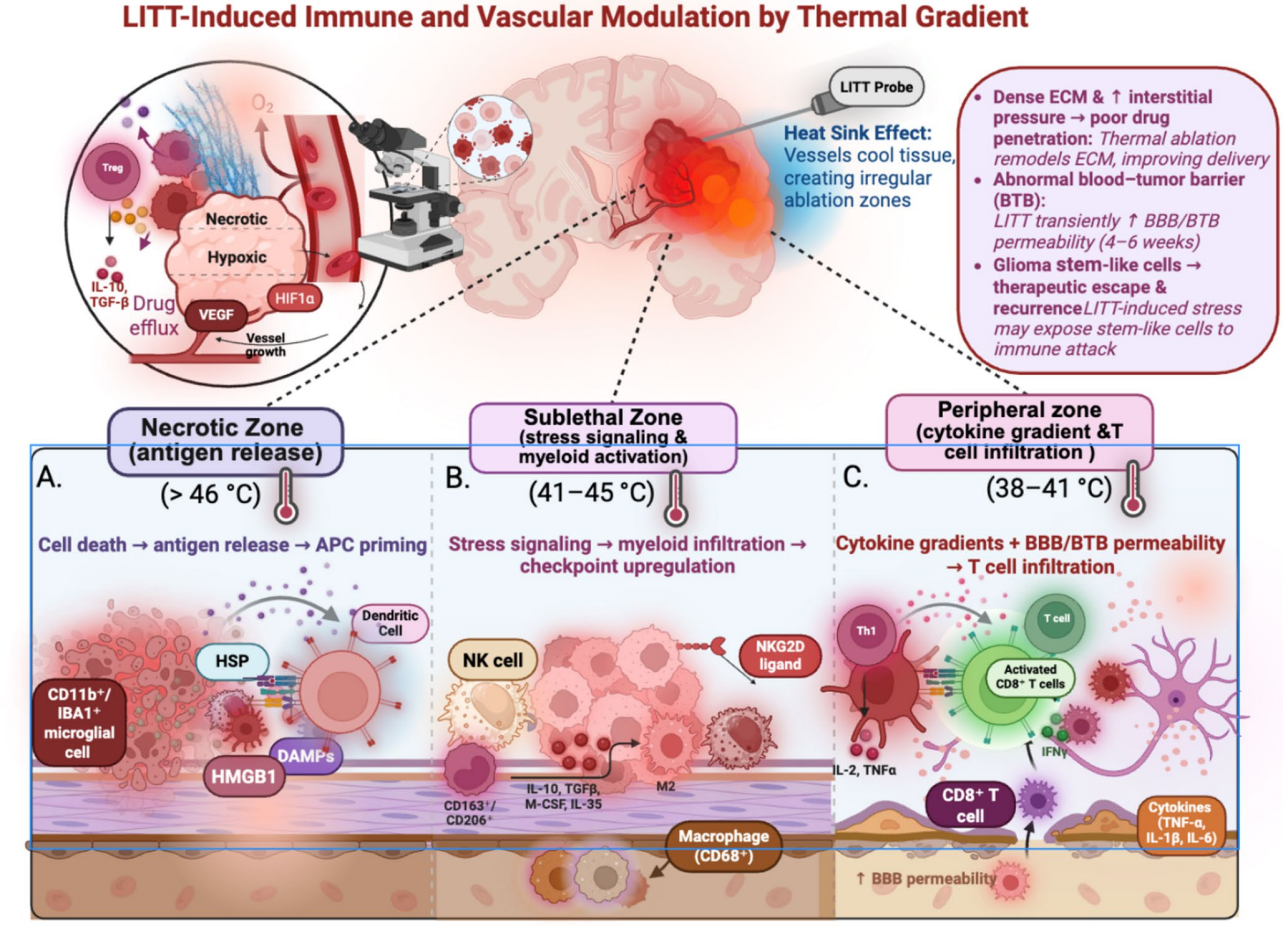
LITT-induced immune and vascular modulation by thermal gradients in the setting of GBM. Laser Interstitial Thermal Therapy (LITT) generates three concentric temperature zones with different biological effects on glioblastoma. (**A**) Necrotic zone (> 46 °C): coagulative necrosis of glioma cells induces immunogenic cell death with the release of damage-associated molecular patterns (DAMPs; HSPs, HMGB1) that stimulate dendritic cells and CD11b⁺/IBA1⁺ microglia to present the antigen. (**B**) Sublethal zone (41–45 °C): heat stress causes upregulation of NKG2D ligands and PD-L1, recruitment of macrophages (CD68⁺), and temporal polarization from pro-inflammatory M1-like phenotypes (MHCII⁺) to immunosuppressive M2-like phenotypes (CD163⁺/CD206⁺) with the production of IL-10, TGF-β, M-CSF, and IL-35, hallmark of systemic immunosuppression characteristic of such CNS neoplasm. Synergistic immunotherapy is administered through NK cell activation and checkpoint upregulation. (**C**) Peripheral zone (38–41 °C): moderate hyperthermia generates cytokine gradients (TNF-α, IL-1β, IL-6, IFN-γ), increases BBB permeability, and permits entry of activated CD8⁺ T cells and Th1 effectors. Collectively, these regions demonstrate how LITT reconditions the GBM tumor microenvironment through promising orchestration of antigen release, myeloid activation, checkpoint signaling, and acute vascular permeability into a therapeutic window that can be used for systemic delivery of therapy. Schematic, not to scale. Boundaries are illustrative; actual thermal maps are irregular and influenced by heat-sink effects near vessels and CSF. Distances are exaggerated to convey concepts. Figures were made using Biorender, Accessed August 28th, 2025

In LITT, real-time MR thermometry provides accurate, dynamic temperature mapping to keep tissue within the therapeutic window; temperatures drop sharply with distance from the fiber (around 10 °C/mm), creating a central coagulative core and graded peripheral heating. At levels above the therapeutic range, brain injury results from protein denaturation, BBB breakdown, vasogenic edema, and mitochondrial dysfunction—effects that highlight the importance of careful thermal limits and perioperative monitoring [20]. LITT induces necrosis through thermal coagulation, producing three concentric zones defined by temperature gradients (Fig. 1). At the core, temperatures exceeding 46 °C cause rapid irreversible damage, with necrosis at 50–100 °C. The transitional zone (41–45 °C) causes sublethal stress with reversible mitochondrial and DNA damage if brief, but prolonged exposure induces apoptosis. The outer zone (38–41 °C) induces hyperthermia, where APCs release proinflammatory mediators and enhance immune activation. Histology shows central necrosis, intermediate macrophage-rich tissue, and outer edema, with viability increasing at greater distances and lower temperatures [4, 21].

Beyond cytoreduction, LITT may convert ‘cold’ gliomas into more immunologically active ‘hot’ tumors. In the sublethal zone, hyperthermia upregulates heat shock proteins (HSPs), acting as DAMPs. These stimulate NK cells and activate APCs, enhancing cytokine release and antigen presentation [4]. The heightened cytokine release and antigen presentation may enhance T-cell-mediated immune responses against residual tumor cells. In one GBM case, post-LITT biopsies showed increased CD8⁺ T cells, IBA1⁺ macrophages, and PD-L1 upregulation, unlike radiation-treated tissue which showed minimal infiltration [22]. These findings suggest that LITT transforms the GBM microenvironment from an immunosuppressive, poorly infiltrated state into a more immunologically active niche.

Thermal stress also promotes myeloid infiltration: M1 macrophages express CD68/MHC II, while M2 co-express CD68 with CD163, CD204, or CD206. At approximately day 14 after LITT, the lesion exhibits a granulation rim with dense CD68⁺ macrophages and CD45⁺ infiltrating leukocytes surrounding the necrotic core [23]. The temporal dynamics of macrophage polarization after LITT remain poorly understood, warranting further study. Clarifying when the TME shifts from pro-inflammatory to immunosuppressive could optimize checkpoint inhibitor timing. If M2-like macrophages dominate later, combining LITT with CSF1R inhibitors or CD206 antibodies may sustain antitumor immunity [24]. Other thermal injury models suggest defined timelines: in trauma and burn patients, M1 peaked at 7–14 days before declining, while M2 appeared by day 2 and stayed elevated for weeks [25]. The benefit of LITT-induced immune activation depends on response magnitude and timing. Early inflammation may support immunity, especially with immunotherapy, but chronic inflammation or M2 accumulation may promote suppression and progression.

## Thermal dynamics of LITT and the heat sink effect

For LITT to be effective, cytotoxic temperatures must be achieved throughout the tumor. Thermal gradients and tissue perfusion influence heat distribution during ablation [20]. Temperatures decrease rapidly as the distance from the laser probe increases, leading to a coagulative necrosis core surrounded by sublethal heating zones [20, 24]. Across histopathology and MR-thermometry studies, the transitional ‘sublethal’ rim is narrow—generally about 1–2 mm between the necrotic core and adjacent parenchyma—and rarely exceeds 2 mm with standard clinical parameters. Its thickness varies with energy delivery, probe/cooling design, and local perfusion or heat-sink effects, but it remains confined to a few millimeters in both preclinical and clinical series [25]. This steep, controllable thermal gradient underpins LITT’s spatial precision: it produces predictable concentric biological effects rather than a single uniform damage zone.

A major limitation is the “heat sink effect”, where blood vessels dissipate heat, cooling tumor tissue and reducing ablation effectiveness in perfused areas. This is most relevant in highly vascular regions, such as the peritumoral vasculature. Thus, it may cause suboptimal ablation in perivascular areas, leaving viable tumor cells [26]. Residual cells may drive recurrence, particularly in perivascular niches that evade ablation and immune surveillance.

Furthermore, inadequate heating in these areas might hinder the release of DAMPs and other signals that stimulate the immune system, thereby limiting LITT’s ability to make the TME more immunogenic and immunoresponsive. To address this challenge, intraoperative MR thermometry can identify under-heated regions in real time, allowing neurosurgeons to adjust the probe position, power levels, or ablation time [27]. To further assist surgeons, preoperative vascular imaging can help identify heat sink-prone zones, enabling the optimization of probe trajectory planning and ablation strategies that account for vascular anatomy [28]. Finally, emerging strategies to mitigate heat sink effects also include the use of dual-fiber LITT systems, which allow for greater spatial coverage and thermal overlap in perfusion-rich regions. Experimental studies in both ex vivo and in vivo animal prostate models have shown that simultaneous activation of two closely spaced fibers produces more uniform and complete ablation volumes than serial single-fiber treatments, even in highly vascularized tissue. These findings underscore the potential of dual-fiber configurations to overcome localized heat dissipation and improve ablation efficacy in intracranial applications [29].

## Blood-brain barrier disruption and drug delivery

**Fig. 2:**
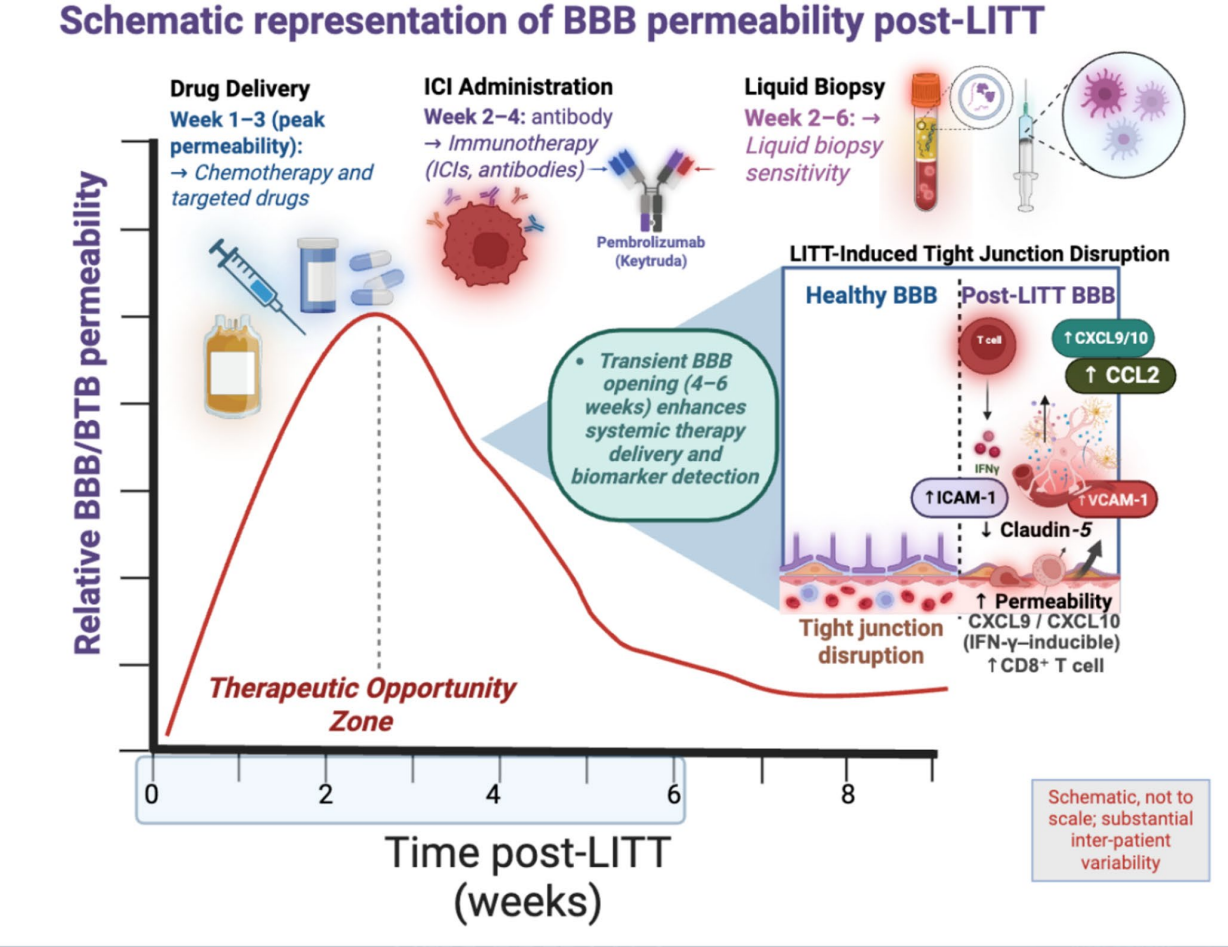
Transient BBB disruption and therapeutic window following LITT. Laser interstitial thermal therapy (LITT) causes transient elevation in blood–brain barrier (BBB) permeability, which lasts for about 4–6 weeks,. This window creates a therapeutic window for better chemotherapeutic and targeted agent delivery and, and antibodies during periods of peak antigen shedding and checkpoint upregulation (weeks 2–4), and biomarker shedding to the circulation that increases liquid biopsy sensitivity (weeks 2–6). Mechanistically, LITT amplifies tight junction disassembly with claudin-5 downregulation but CAM-1, VCAM-1, CCL2, and IFN-γ–inducible CXCL9/10 upregulation to facilitate immune cell migration through the weakened barrier. These vascular and immunologic changes cumulatively create a synergistic platform for systemic delivery of therapeutics and diagnostic monitoring of GBM tumor microenvironment. Figures were made using Biorender, Accessed August 28th, 2025. Figure 2 is a schematic illustrating evolving models of BBB permeability post-LITT. Early human data show an acute rise resolving by around 4–6 weeks, whereas recent controlled DCE-MRI shows a delayed, sustained increase (days 15–45), highlighting inter-patient variability. These windows are schematic and based on limited human DCE-MRI permeability data and early translational reports of checkpoint upregulation after thermal injury; inter-patient variability is substantial, so timing should be personalized and viewed as hypothesis-generating

LITT also temporarily disrupts vascular integrity. Heating to 38.5–42 °C promotes the temporary disruption of the BBB, enhancing drug delivery and immune cell access to the tumor site [4]. The BBB, a semipermeable border that restricts the entry of drugs into the central nervous system, is the main obstacle for systemic therapies to achieve therapeutic concentrations in GBM. However, GBM often forms a heterogeneous and partially permeable blood-tumor barrier (BTB), characterized by abnormal endothelial junctions, disorganized astrocytic end-feet, and variable expression of efflux transporters [30]. LITT-induced thermal damage can temporarily increase BTB permeability and improve intratumoral delivery (Fig. 2). Notably, the increase in BBB permeability following LITT can last for about 4 to 6 weeks [31]. Pioneering human research using DCE-MRI and serum BSE showed an immediate increase in peritumoral permeability after LITT that gradually declined toward baseline over approximately 4–6 weeks [32]. A newer, controlled DCE-MRI study found no day-1 increase but a robust, sustained permeability rise at days 15–45, refining the therapeutic window toward a delayed subacute phase [33]. Preclinical mouse models have shown that heating to 43 °C causes tight junction proteins, like claudin-5, to be downregulated and disorganized, which correlates with increased BBB permeability. Another way LITT enhances BBB permeability is by increasing transcellular transport, which is typically low in brain endothelial cells [34].

Other methods, such as low-intensity focused ultrasound (LIFU), can also temporarily open the BBB by using transcranial ultrasound waves and intravenous microbubbles to loosen endothelial junctions via non-thermal mechanical effects; however, focal heating can occur depending on acoustic parameters [35]., Unlike LITT, which can maintain BBB permeability for 4–6 weeks, the effects of LIFU typically last only a few hours, making LITT a more suitable choice when a longer therapeutic window is needed for systemic therapy delivery.

This prolonged BBB permeability following LITT has been exploited in both preclinical and clinical contexts to enhance treatment efficacy. Preclinical studies in mice with GBM have shown that combining LITT with doxorubicin results in higher doxorubicin levels in brain tissue, a significant reduction in tumor growth, and a 71% increase in median survival compared to controls [34]. In addition to increasing BBB permeability for existing drug formulations, special encapsulated drug forms could also be exploited by LITT. Microbubbles are lipid, protein, and polymer spheres that can be filled with drugs. When exposed to external energy sources, especially ultrasound, they collapse and help with drug delivery. Although microbubbles are most commonly used in conjunction with LIFU, emerging preclinical research suggests that they can be combined with other thermal therapies, such as LITT. Using ultrasound-stimulated microbubbles with hyperthermia proved more effective at decreasing tumor size than either method alone. Additionally, incorporating microbubbles during laser therapy significantly raised local temperatures and enhanced tumor cell death in vitro. However, no current clinical trials or established guidelines support the use of microbubbles combined with LITT in humans [36].

Early-phase trials suggest that combining LITT with immune checkpoint inhibitors may yield survival benefits in select GBM patients, likely due to increased antigen release and immune cell infiltration that occurs during BBB disruption. The recent Phase I/II study of LITT plus pembrolizumab in recurrent, bevacizumab-naïve high-grade glioma reported that four out of nine patients achieved survival of at least 30 months, with no dose-limiting toxicities observed. These findings support the notion that LITT could serve as an in situ vaccine, supporting the release of tumor antigens and promoting immunogenic cell death. This effect is believed to occur due to the release of a wide range of tumor-associated antigens and damage-related molecular patterns (e.g., heat shock proteins, HMGB1) into the tumor microenvironment, which encourage dendritic cell activation and subsequent T-cell priming, thereby strengthening anti-tumor immune responses [30]. At the same time, the increased permeability of the BBB improves the infiltration of immune cells and antibodies. Furthermore, the increased BBB permeability may also be used to facilitate viral and gene therapies. The STARLITE trial (NCT06428045), involves combining LITT with antiretroviral therapy (abacavir, lamivudine, ritonavir) for patients newly diagnosed with unresectable high-grade glioma. This trial demonstrates that LITT could enhance the delivery of specific agents to tumors.

Furthermore, LITT could improve the sensitivity of liquid biopsies, an underexplored diagnostic adjunct in this context. By temporarily increasing BBB/BTB permeability and causing tumor cell lysis, LITT may increase release of circulating biomarkers (ctDNA, EVs) into the bloodstream and thereby improve detection after the procedure [30]. While antigen/EV bursts probably happen right after ablation, the subacute phase of ongoing—but variable—permeability (~ 2–6 weeks) might extend a noninvasive monitoring window for response assessment or minimal-residual-disease surveillance; this should be seen as complementary to tissue biopsy during LITT, not a substitute. Given the transient signal and assay limits, prospective serial sampling (baseline, 24–72 h, and weeks 2–6) and clinical validation are still necessary before routine use [37].

A parallel principle—using a localized physical modality to sensitize gliomas to systemic therapy—appears in other emerging strategies. For example, in preclinical studies, noninvasive cold atmospheric plasma (CAP) can make glioblastoma cells more sensitive to temozolomide and significantly slow intracranial tumor growth in animal models [38, 39].While the increased BBB permeability caused by LITT provides many valuable therapeutic opportunities, it also introduces potential risks. The most common complication is cerebral edema, which results from increased permeability leading to vasogenic fluid leakage that can last for two to six weeks post-LITT. Another concern is hemorrhage, as thermal damage to blood vessels may cause procedural bleeding [5]., patients might experience seizures, likely due to thermal irritation or edema-related pressure, and neurological deficits, especially when lesions are close to the eloquent cortex (however the latter is a risk in surgical re Additionally section too). Like any intracranial procedure, LITT carries a risk of infection because it involves inserting a probe into the brain through a burr hole, although this foreign body is temporary during the procedure and is subsequently removed. Therefore, accurate targeting and perioperative monitoring are crucial during LITT. Despite these risks, current clinical and preclinical data indicate that the benefits of LITT outweigh its potential complications when patients are carefully selected and closely monitored [40].

Importantly, BBB/BTB opening is bidirectional: during the subacute period—most evident in weeks 1–3 with recovery around 2–4 weeks—permeability increases not only to therapeutics but also to circulating cytokines, infectious mediators, and neurotoxic medications [21]. Practical safeguards include thorough medication reconciliation focusing on CNS-penetrant or neurotoxic agents, using non–enzyme-inducing antiseizure drugs (e.g., levetiracetam), and implementing protocolized corticosteroid management for edema. Bevacizumab should be reserved for steroid-refractory cases or when edema worsens due to recent radiotherapy. Vigilant monitoring for infection and neurocognitive changes, along with early interval MRI to observe evolving edema, hemorrhage, or progression, is recommended during this period [41]. Currently, prospective, standardized post-LITT monitoring pathways are limited; existing guidance primarily relies on expert consensus and retrospective data [7].

### Patient selection and clinical indications

Across meta-analyses and large series, LITT is most appropriate for deep, eloquent, well-defined, small-volume lesions—typically less than 3 cm—in patients who are poor candidates for craniotomy or who have recurrent HGG/GBM or brain metastases after prior treatment. Favorable candidates include deep-seated brain metastases that grow after prior SRS or appear as suspected radiation necrosis, for which LITT provides a minimally invasive option with good local control and safety. Relative contraindications include large size (>3–4 cm), proximity to critical neurovascular or CSF spaces (risk of high heat-sink/thermal injury), multifocal or diffuse disease, uncontrolled bleeding disorders, and low functional status (e.g., poor KPS), all linked to worse outcomes or more complications [10]. Reported failures often involve incomplete ablation in highly vascular or irregular tumors and baseline frailty, with pooled Progression Free Survival, Overall Survival, and complication rates varying by tumor type and group [42]. These criteria offer a practical framework for counseling and comparing LITT with other local treatments.

Compared to open craniotomy, LITT is associated with a shorter hospital stay (median approximately 1.9–3.2 days), although preoperative frailty and larger tumor volume can predict longer stays. Routine ICU admission is rare, and overall hospital costs seem similar to craniotomy (average around $124,225 in a national sample), with limited data on long-term cost-effectiveness [43]. Patient-reported outcomes (e.g., FACT-Br, KPS) are generally stable or improved after LITT, but 1-year readmission rates are notable—often due to tumor progression—and long-term PRO data are still limited [5, 6].

### Clinical evidence for LITT as a therapeutic potentiator

Although clinical work remains in early stages, several studies have evaluated LITT as a platform to enhance systemic therapies. In humans, a pilot phase I/II study (NCT01851733) tested LITT followed by weekly low-dose doxorubicin (20 mg/m² for 6 weeks) in 30 evaluable patients with recurrent GBM. The study initially enrolled a late cohort (6–8 weeks post-LITT) and then randomized subsequent participants 2:1 to early (≤ 72 h) versus late (6–8 weeks) dosing. Primary goals focused on BBB permeability and feasibility; survival analyses were exploratory and compared with historical controls rather than a concurrent control group. Signals for longer overall survival were observed at the cohort level (numerically favoring later timing), but PFS was not improved. Since the study was not powered for definitive efficacy, results should be interpreted with caution.

An ongoing phase I/II study (NCT03277638) is specifically testing pembrolizumab administered at different timepoints relative to LITT (− 7 days pre-LITT, + 14 days, or + 35 days), with primary endpoints of safety/tolerability and determining the optimal timing, and secondary endpoints of ORR, PFS, and OS. A three-patient case series of recurrent IDH-wild-type GBM reported radiographic and clinical responses to LITT followed by pembrolizumab, illustrating feasibility and a potential immunologic signal in humans [44]. For newly diagnosed HGG, a prospective study showed that starting chemoradiation within approximately one week after biopsy and LITT was feasible and safe, supporting the integration of LITT into upfront treatment schedules pending efficacy data [45]. Together, these early studies are hypothesis-generating and point to timing as a critical variable; larger, controlled trials are needed to determine optimal sequencing and patient selection.

### Comparison with SRS and TTFields

Mechanistically, LITT causes immediate thermal cytotoxicity with immunogenic cell-death signals (DAMP release, PD-L1 upregulation, CD8⁺/myeloid infiltration) and a predictable, transient BBB/BTB opening that peaks within weeks 1–3, creating a short, bidirectional window for drug delivery and toxicity monitoring [21]. Stereotactic radiosurgery (SRS) results in delayed DNA-damage–mediated cell death with immunologic effects that develop over weeks; changes in vascular permeability are less consistent and often linked to radionecrosis rather than a controllable delivery window [46]. Tumor Treating Fields (TTFields) exert antiproliferative effects by disrupting mitosis and can activate innate immune pathways (e.g., cGAS/STING, AIM2), but any effects on the BBB appear modest and are not well defined over time compared to LITT [47]. These differences suggest distinct “combination windows”: LITT favors schedule-sensitive pairing with systemic agents during subacute permeability, while SRS and TTFields are generally sequenced empirically or administered concurrently, respectively [48]. Direct head-to-head data on permeability and immune signatures remain limited, highlighting the need for comparative translational studies [31, 49].

## Limitations

This article is a narrative, hypothesis-generating review rather than a systematic review or meta-analysis; we did not conduct a structured search, risk-of-bias assessment, or quantitative synthesis. Many claims regarding immune modulation and blood–brain barrier (BBB) dynamics after LITT originate from preclinical models or small, early-phase human studies and may not apply to the heterogeneous, heavily pre-treated GBM population. Apparent clinical signals (e.g., drug-delivery enhancement, immune “warming”) may be influenced by selection bias, confounding, or publication bias, and randomized data are still lacking. The heat sink effect and perfusion heterogeneity can cause patchy, asymmetric thermal distributions and incomplete ablation in a significant subset of cases; prospective quantification and standardized reporting are limited. Our figures are schematic and designed to illustrate biological concepts—not to show patient-level variability or precise spatial immuno-thermal relationships. Importantly, BBB disruption is bidirectional: while it may improve therapeutic delivery, it could also permit entry of systemic cytokines, toxins, or medications with neurotoxic potential; best-practice monitoring or prophylactic strategies during this window have not been established. Comparative effectiveness versus other local options (e.g., stereotactic radiosurgery, tumor treating fields), as well as cost, quality-of-life, and the long-term effects of repeated BBB disruption, remain insufficiently explored. Future research should focus on randomized or well-controlled prospective trials, standardized MR-thermometry and permeability measurements, refined patient selection criteria, and clear safety protocols for the post-LITT period.

## Future directions

LITT is redefining the therapeutic landscape for glioma management by functioning not only as a cytoreductive tool but also as a potent modulator of the TME. Through precise thermal control, LITT induces spatially distinct zones of immune and vascular modulation, triggering the release of DAMPs, enhancing local immune activation, and transiently disrupting the BBB. These effects synergize to improve both the immunogenicity of gliomas and the intratumoral delivery of systemic therapies. It may also be possible to blunt heat-induced cytoprotective programs (e.g., HSPs) to turn transitional-zone injury into complete ablation, a concept that warrants preclinical testing.

Importantly, LITT enables targeted ablation in anatomically complex or surgically inaccessible brain regions, offering a minimally invasive alternative to open resection with lower neurological risk. The therapy’s ability to convert immunologically “cold” gliomas into more responsive “hot” tumors opens the door to novel combinations with checkpoint inhibitors and selected chemotherapeutics or gene-based treatments [50]. Moreover, the 4–6 week window of BBB disruption presents an underutilized opportunity to enhance liquid biopsy sensitivity and deliver otherwise impermeable agents directly to the tumor site.

Still, critical questions remain. The temporal dynamics of macrophage polarization, the long-term consequences of thermal immune activation, and the optimal timing for adjunctive treatments require further investigation. Understanding these processes will be essential to fully leverage LITT as a platform for immune priming and multimodal therapy.

Looking ahead, future research should build on current progress by standardizing advanced vascular imaging and thermal modeling for individualized LITT planning, expanding immune profiling use for patient stratification, and refining timing and synergy of systemic therapies during the 4–6 week BBB window. Comparative studies with other BBB-opening modalities, such as focused ultrasound, will also be essential to define LITT’s unique therapeutic niche.

## Data Availability

No datasets were generated or analysed during the current study.
